# Accountability as a virtue in medicine: from theory to practice

**DOI:** 10.1186/s13010-023-00129-5

**Published:** 2023-03-22

**Authors:** John R. Peteet, Charlotte V.O. Witvliet, Gerrit Glas, Benjamin W. Frush

**Affiliations:** 1 Department of Psychiatry, Harvard Medical School, Boston, USA; 2 Psychology Department, Hope College, Holland, MI USA; 3 Faculty of Humanities, Department of Philosophy, Vrije Universiteit, Boelelaan 1105, 1081 HV Amsterdam, The Netherlands; 4 Internal Medicine and Pediatrics, Vanderbilt Medical School, Nashville, USA

**Keywords:** Accountability, Virtue, Accountability as virtue, Professionalism, Medical education, Healthcare delivery

## Abstract

Accountability is a norm basic to several aspects of medical practice. We explore here the benefits of a more explicit focus on the virtue of accountability, which as distinct from the state of being held accountable, entails both welcoming responsibility to others and welcoming input from others. Practicing accountably can limit moral distress caused by institutional pressures on the doctor patient relationship. Fostering a mindset that is welcoming rather than resistant to feedback is critical to enhancing a culture of learning. Analysis of failures of accountable practice offers opportunities for improving the delivery of clinical care.

## A conceptual description

Accountability is an important, if implicit, value in medicine. Yet the distinction is not often made between being held accountable and the virtue possessed by one who *embraces being accountable*. While accountability understood as a characteristic of relationships in medicine is important, we argue that the *virtue* of accountability, understood as a specific character disposition, is prior to and required for accountable relationships to form and function effectively in the domain of healthcare. We suggest here that, absent a robust commitment to accountability as a virtue, physicians and other healthcare practitioners would be unable to effectively serve the patients under their care, the practitioners with whom they work, and the institutions which employ them.

In order to understand why accountability proves so central to sound medical practice (which we take to be the broader construct of healthcare delivery beyond the practice of allopathic medicine as a single discipline), it is first important to grasp how accountability has traditionally been construed. In a seminal paper on accountability in health care, Linda and Ezekial Emmanuel [1] described three components of the concept – loci, or the parties involved (*who* is accountable, and *to whom*), domains (*what* they are accountable for), and procedures (*how* parties are held accountable). The details of these procedures, they suggested, differ depending upon the operative model at play in health care – whether professional, economic and/or political.

The structures governing professional accountability in healthcare have since been clarified further by Gerrit Glas [2], who distinguishes among differing functions of norms or values in medicine, and clarifies the relationship between values and virtue. Glas argues that *qualifying* moral norms/principles/values focus on the nature and purpose of the practice, while *foundational* principles and insights are those on which the practice is based (usually scientific, technological expertise, insight into therapy, engineering and the like), and *conditioning* norms are the legal, administrative, institutional and economic conditions that allow practitioners to fulfill their role. While values refer to things in life we deem worthy and valuable, norms are the standards which we are supposed to enact in order to attain the things in life we deem to be valuable. The act of adhering to or exhibiting certain values in our lives is termed ‘norm-responsive’ (or value-oriented/guided) behavior. Norm-responsive behaviors, in turn, become virtues when they are internalized throughout one’s upbringing, education, and training. This process of internalization implies that they become a durable part of who a person is in the role they are supposed to fulfill in the future.

Glas furthermore calls attention to the risk of overemphasizing either the micro (patient care), meso (administration, financial) or macro (regulatory and certification) spheres in which these norms operate, and of allowing foundational or conditioning norms to function as qualifying ones [3].

Ultimately, a lack of clarity on how, and to whom, healthcare practitioners ought to be accountable has important downstream consequences. For example, the risk of conflating qualifying and conditioning norms which govern accountability is demonstrated by the Emmanuels’ prescient warning that “portraying physicians as caring professionals while forcing them to act as economic producers … will ultimately discredit the entire practice of medicine and sow distrust and cynicism that cannot easily be overcome.” [[1], p. 238]. Put another way, the authors warn against the tendency of large institutions to structure physicians’ practice such that they are ultimately accountable for financial considerations, rather than the health and well-being of individual patients. The term “burnout” has since been applied to the resulting demoralization and sense of moral injury that occurs when professionals violate their values, which is more likely to occur as economic or institutional norms supersede the moral norms of the healthcare provider relationship.

Clinical medicine, whether practiced well or poorly, ineluctably consists of the actions and experiences of individual patients interfacing with individual healthcare practitioners, and communication among individuals on the healthcare team. Despite the tendency to view issues such as healthcare quality, patient outcomes, or even burnout, as functions of populations, the moral foundation of medicine ultimately consists in the interactions between individuals. Thus any goal of understanding these broader issues in healthcare also must take seriously the roles, practices, and values of individuals in addition to systems. Understanding accountability as a virtue that might be modeled, taught, learned, and practiced highlights what kind of persons clinicians need to be in order to care well for their patients, form effective teams, and ultimately allow for the flourishing of the broader systems in which they operate.

### The practice of accountability in medicine

While a sound conceptual understanding of accountability is important, accountability as a virtue holds little weight in medicine unless it is intentionally cultivated and *practiced* (given that a virtue rather than a duty based approach emphasizes the practical, educational prerequisites of acquiring good habits) in ways that promote flourishing. Accountability, then, warrants not just attention but also action in order to move from theory to practice [4]. In contrast to the state of being held accountable, the *practice* of accountability as a virtue undertaken by individual moral agents, as addressed by Witvliet and colleagues [5], page 2, entails:

(a) welcoming being accountable *to* others across relationships with others to whom one rightly owes a response–both in receiving capable, sound input of trusted persons and in providing transparent explanations of their own decisions and actions; and (b) being willingly accountable *for* one’s own attitudes, thoughts, emotions, and actions–working to improve or correct one’s responses for a positive impact. Welcoming accountability to others for fulfilling one’s responsibilities to them requires practical wisdom to neither reflexively conform to nor disregard others’ requests or expectations. As such, accountable practice requires clarity about what is owed to whom, empathy in order to appreciate the expectations of the other, and self-regulation to respond appropriately. A culture of accountability is one that actively seeks to instill this clarity, empathy, and self-regulation and thus fosters accountable practice.

Consider the contrast between a welcoming and a resisting mindset toward supervisory feedback [6], a practice which is central to both medical training and practice within medical institutions. A welcoming mindset (1) values feedback as beneficial, useful, growth-producing and equipping for one’s future, (2) values the supervisor as a person whose perspective and experience can help one grow in competence, and (3) values oneself as able to learn from, adapt to, and benefit from the feedback. By contrast, a resistant mindset (1) devalues such feedback as a hassle or burden that is potentially pointless, inconvenient, annoying, frustrating, and difficult, (2) devalues the supervisor’s perspective and input as unnecessary, and (3) devalues change in oneself, instead focusing on how one already knows one’s own abilities and how to work, preferring not to change. Whereas a welcoming mindset embraces learning and growth, a resistant mindset neglects this. A welcoming mindset inclines one to regard the supervisor as a worthy person and engage in empathic perspective-taking; by contrast, a resistant mindset works against the perspective-taking so important for empathic connection and learning another important point of view. A welcoming mindset emphasizes one’s capacities for self-regulation which is essential for responsibly modulating one’s attitudes, thoughts, emotions, and actions in light of feedback; in contrast, a resistant mindset undercuts self-regulation and responsible change.

Contemporary healthcare acknowledges, if only implicitly, the importance of accountable practice as outlined by Witvliet and colleagues, through existing methods for reviewing and improving care. Morbidity and Mortality (M and M) conferences, Quality Assurance and Improvement (QA/QI) efforts, and the systematization of supervision in clinical practice all attest to this. An emphasis upon the centrality of accountability is also explicitly built into medical training; the Accreditation Council for Graduate Medical Education (ACGME) names accountability as an element in the core competence of professionalism, by which medical trainees are regularly evaluated. However, whether the term “accountability” refers to the condition of being held accountable or to welcoming accountability is generally left undefined, and no consensus exists on how to work toward cultivating the virtue.

## Exploration of failures of accountability

One of the clearest ways to recognize the importance of accountability as a virtue affecting healthcare is to note when its absence adversely affects patient care, practitioner experience, or the functioning of healthcare systems. To explore this, we consider four examples of failures of accountability extending from the micro to the macro level, in order to better understand and then address the factors involved.

1. Rita Charon’s seven minute video “Intern Progress Note” [7] features a harried medical intern who is responsible for an increasing number of patients despite the failures of the system to support the intern in performing the tasks involved. The medical intern is clearly attempting to practice accountably to patients, but the patients’ needs exceed the intern’s capacity. Physicians in outpatient primary care also often find themselves frustrated if not overwhelmed by the multiple expectations crowding out time for the role they entered medicine to play [8, 9]. One wonders why no one offers to help. What accounts for an apparent lack of mutual accountability to and for one another?

Potential contributing factors include a traditionally hierarchical medical culture, where superiors believe their juniors must negotiate competing loci of accountability without assistance; stress on co-practitioners which constrains their ability to act more generously or collaboratively; and almost certainly, larger competitive economic or political forces which de-prioritize the experience of practitioners and individual patients alike in the name of financial or temporal expediency. Practicing accountably would require clarification of priorities (to whom is the clinician primarily accountable, and for what?), recognition of collective responsibility (to whom and for what?) and, accordingly, a shift from an error-focus toward a systems approach to safety, quality, and self-learning in organizations [10].

2) A progressive, young health care organization contracts with payors to care for a population of people who live with disabilities in the community, aiming to minimize costly hospitalizations. The organization’s CEO makes an executive decision to cut the staff of the clinic team because it loses money relative to the team in the field who visits clients in their homes. Clinicians request a meeting with the CEO, objecting that no one had consulted them, or had offered to examine how their patients experienced the quality of their care. One might ask what accounts for this failure to involve clinicians in discussion of their shared accountability to patients and to the financial viability of the organization.

It seems possible that the CEO inappropriately applied an economic and political model in lieu of a professional model, in such a way that what was owed patients and staff, including respect for their dignity and worth, was de-prioritized, perhaps out of a concern to responsibly steward organizational resources. It could be that while these factors were indeed considered, there was a failure of healthy, transparent communication. It could also be that the CEO was insufficiently aware of the importance of the organization’s social responsibility [11, 12] and the risk of succumbing to market driven competition. This lack of awareness could relate to the CEO’s own vulnerability to a performance-based identity that implicitly or explicitly measured their (and the enterprise’s) worth comparatively, rather than in relation to the moral context within which they are accountable—to both people within, and outside of, the organization.

Here, accountable practice in the micro-sphere might entail questioning whether the clinic staff was legitimately accountable to the CEO for productivity metrics if these measures conflict with those mandated by their relationships with patients and their obligations to provide professional care. At the meso- and macro-level of organization of care it could (again) be asked whether both the medical staff and the CEO were sufficiently aware of what it means to be socially responsible as an organization.

3) An academic medical center recruits a division chief based solely on research reputation, without including practice-oriented members of that division as part of the search committee. Within months of arriving, the new chief enacts arbitrary and dictatorial changes to staff roles and requirements that result in multiple complaints, resignations and concerns about deteriorating clinical care. The hospital leadership then confines the new hire to the lab and appoints an interim chief.

In this case, an apparently narcissistic personality with limited capacity for empathy and self-regulation with humility—which are intrinsic to the practice of mutual accountability—was initially selected by leaders of an institution. These leaders were concerned with the organization’s competitive reputation as a comparative measure of its worth, rather than prioritizing the moral context of professional accountability. Here, exercising the virtue of accountability at the institutional level would involve recruiting a chief with different character dispositions—such as empathy, humility, and self-regulation—who was capable of supporting and engaging others with relational responsibility, rather than dominance that sought others’ servile acquiescence.

4) A patient experiences severe back pain beginning during physical therapy, five days following a lumbar laminectomy. The original neurosurgeon had taken a personal leave due to work stress. After some difficulty in securing an appointment, the patient was readmitted to the hospital where the surgery was performed. On meeting the covering surgeon who had a burgeoning caseload, the patient expresses appreciation for being accepted as a patient. In response, the covering neurosurgeon, who has no time for more than a glance at the imaging, dismissively remarks, “I didn’t accept you, and your imaging is probably fine,” discharging the patient before being able to walk. The patient requires admission to another hospital where a careful review of the MRI shows nerve impingement.

One wonders here not only about character, but also work culture and the stress imposed on the covering neurosurgeon, whose professionalism was undermined by an excessive caseload and the personal leave of a colleague due to work pressures. For the covering neurosurgeon to show accountability as a virtue would have meant providing care the patient was due by carefully reviewing the evidence related to the patient’s symptoms and concern, as well as addressing aspects of the work culture that could be improved to de-escalate stress and promote adequate supports for medical staff, patient appointment times, and transparency.

## Toward cultures of accountability in medicine

These scenarios show how factors such as hierarchical cultures, personal narcissism, deficient communication, institutional competition, and work stress can combine to undermine the practice of accountability. They suggest to us a need for the following to work toward accountable practice:

1. A systematic root cause analysis of failures of accountable practice is important. We suggest beginning with an examination of the responsibilities and obligations of the relevant stakeholders, including how these are being met or missed, and proceeding to consider factors such as work overload or a lack of support that could be making requisite components of the virtue—transparency, empathy or self-regulation—difficult. In their study of accountability in five hospital systems, Aveling et al. [13] found that various forums of accountability such as Morbidity and Mortality (M and M) conferences could be helpful. However, their effectiveness was also conditioned by the vested interests of senior staff and had the potential to become punitive. Their analysis highlighted the process as a responsibility shared by all individuals involved.
2. More explicit discussion of the core values of health care organizations (including at the level of their governing boards) are valuable to guard against lapsing into comparative valuing with the pressure that creates. Performance-based identities at the organizational level can inadvertently place productivity and/or reputation ahead of quality patient care, thereby reversing the order between relevant normative principles highlighted in Glas’ work. This reversal occurs when principles that shape the conditions for the practice of healthcare (such as efficiency and maximizing productivity) transform into principles that qualify that practice. The resulting culture of stress and demoralization elevates the risk of moral injury occasioned by decisions driven by misplaced priorities at odds with more foundational values.
3. Heightened awareness of the need of a shared notion of corporate responsibility goes hand in hand with developing a systems approach to quality, safety, and prioritizing of care. Medical flaws and failures are seldom errors for which only one individual can be held accountable; they also and always deserve to be investigated as expressions of the functioning of the organization as a whole. As the work of Aveling et al. [13] and the example of Dr.Bawa-Garwa [14] suggests, rule-based approaches to achieving a just work culture are less effective than a participatory process in which all individuals create, modify and are subject to the social forces that are an inescapable feature of any organizational system. Glas [3] suggests some possible ways that a physician stressed by intruding forces from meso and macro levels could amplify their reflective space. [Wording is modified for inclusion.] A healthcare professional

“could, for instance, raise [one’s] voice in [one’s] own professional organisation; discuss [one’s] worries with the administrators of the hospital; search for common ground between [oneself], the administrators and patient representatives; take part in the council of hospital employees; participate in advocacy groups; raise public awareness of what is going on in the sector, for instance on social media; or become politically active. Professionalism entails the awareness of and the ability to negotiate about the conditions under which healthcare is delivered.”

These examples underscore the need for the social contract between medical disciplines (e.g., psychiatry) and society to be reviewed and renegotiated on a regular basis [15].

4) Discernment is also important at the individual level to navigate what it means to welcome being accountable to other people and also to one’s highest authorities and ideals—especially in the frequent case of competing claims for loyalty. Aveling et al. [13] found in their study of accountability in five hospital systems that individuals often triumphed in the face of adversity through the exercise of a morally-founded agency, even though it seemed that the conscious choices and actions of individuals were heavily conditioned by strongly reinforced norms and other constraints, some of them deeply institutionally and historically patterned. One’s highest loyalties might be for example to a beloved work community, or to personal and professional ideals, perhaps supported and shaped by reference to one’s faith tradition [16]. This involves both a naming and a rank-ordering of goods for the individual, to place the parties or institutions to whom one is accountable in their proper context. It also may call for transparency to enable discussion and attempts at consensus in cases where values are not shared, as in the case of conscientious objections to providing care. This does not mean that professionals operating within the healthcare context will avoid such dilemmas of accountability—only that they might approach these dilemmas with more clarity when they have done the work of discerning commitments a priori.
5) Identifying and cultivating the requisite ingredients of accountability as a virtue will benefit trainees in developing the virtue. While medical training is typically viewed primarily as a time of informational and technical acquisition, it is in fact better understood as a profound period of moral formation [17, 18]. In order to select for those who might flourish in this intense space, what questions can leaders ask to better recognize state and trait accountability in job applicants? Sample behavioral questions already being suggested to interviewers for use in residency recruitment interviews include:

“Describe a time when you disagreed with an evaluation or feedback you received about your performance. How did you handle the situation? What impact did it have on you? What did you learn?” “Describe a time when you received negative feedback and turned it into an opportunity to make a correction or improvement, or to grow and learn.”

6) It is important to know how important empathy and self-regulation are to the virtue of accountability to others, to identify specific ways individuals may benefit from remediation. The human aversion to negative feedback can be particularly strong for seniors in medicine who have generally enjoyed success amidst the rigors of schooling and training. Remediation and feedback by peers are critical for improved future practice. For example, similar to the way that mentalization training has been developed to enhance empathy in the treatment of individuals with borderline personality disorder, exercises are now being studied which can enhance a welcoming mindset to feedback from others by valuing what one can learn from others’ perspectives and seeing oneself as capable of adapting as needed. Such work points to the value of adopting a welcoming mindset to supervisory feedback, which elevates empathy, self-regulation, and accountability [6].

## Conclusion

We started by making a distinction between the state of being accountable and the virtue of accountability. We argued for the importance of the virtue of accountability to responsibly and competently serve patients, collaborate with colleagues, carry out professional formation, and uphold proper standards of care and professionalism. We discussed failures of accountability in cases that reached from the micro to the macro level, i.e., in the provision of patient care, the operation of the clinical team, the realization of a hospital’s teaching mission, and the system of healthcare delivery. We offered a theoretical frame and examples with practical steps toward both remediation and prevention as well as proactive cultivation of the virtue, ultimately with the goal of improving patient care, practitioner experiences, and the well-working of healthcare systems.

## Data Availability

Not applicable.
